# Psychological flexibility as a moderator of the association between premenstrual dysphoric disorder, depression, anxiety, positive parenting, and negative parenting: a cross-sectional study

**DOI:** 10.1186/s13030-025-00327-x

**Published:** 2025-04-07

**Authors:** Junko Okajima, Isa Okajima

**Affiliations:** 1 Department of Psychology, Rikkyo University, Rikkyo, Japan; 2 Department of Rehabilitation, University of Tokyo Health Sciences, Tokyo, Japan; 3 Department of Psychological Counseling, Faculty of Humanities, Tokyo Kasei University, Tokyo, Japan

**Keywords:** Acceptance and commitment therapy, Psychological flexibility, Negative parenting, Positive parenting, Premenstrual dysphoric disorder (PMDD), Depression, Anxiety, Mothers toddler development

## Abstract

**Background:**

This study aimed to investigate whether psychological flexibility moderates the relationship between premenstrual dysphoric disorder (PMDD) symptoms and depression, anxiety, positive parenting, and negative parenting.

**Methods:**

For this study, a sample of 1,538 menstruating mothers with children aged 0 to 3 years (227 with 0-year-olds, 428 with 1-year-olds, 409 with 2-year-olds, and 424 with 3-year-olds) was assessed utilizing the Premenstrual Dysphoric Disorder Scale (PMDDS), Parental Acceptance and Action Questionnaire (PAAQ), and Hospital Anxiety and Depression Scale (HADS).

**Results:**

The interaction effects between PMDDS and PAAQ scores were found to be significantly associated with anxiety and positive parenting, after controlling for other variables. Notably, higher PAAQ scores were associated with increased positive parenting, even in the presence of worsened PMDD symptoms. Furthermore, psychological flexibility, as measured by the PAAQ, had an independent effect on both depression and anxiety, though no moderating effect was observed.

**Conclusions:**

Interventions aimed at enhancing psychological flexibility may be beneficial for mothers with premenstrual dysphoric disorder who are raising infants and toddlers.

## Background

In 2022, Japan’s birth rate was projected to fall below 800,000 for the first time, exhibiting an accelerating decline, while the number of child abuse cases increased with an additional 11,510 cases reported annually [1]. A prior study indicated that 8.9% of mothers fell into the abusive category, while 30.4% were classified as sub-abusive [2]. These statistics suggest that a significant number of mothers are at risk of engaging in abusive behaviors, thus jeopardizing their child-rearing situations.

Dalton [3] highlighted clinical findings indicating that premenstrual syndrome (PMS) often develops and worsens after childbirth. Premenstrual syndrome is characterized by various mental and physical symptoms, including irritability, depressive mood, fatigue, mood swings, and anxiety. Premenstrual dysphoric disorder (PMDD), a severe form of PMS, presents with more pronounced psychological symptoms, such as mood instability, increased irritability, and heightened nervousness. The prevalence of PMS among women is approximately 18–21% [4, 5], while that of PMDD ranges from 3 to 8% [5–8]. Among Japanese women aged 20–40 years, those who have given birth report significantly more psychological symptoms, such as irritability and depression, compared to physical symptoms [9]. Furthermore, these findings suggest a potential association between PMS and PMDD symptoms and problematic emotional interactions with children [10]. A prior study that conducted a systematic review of cognitive behavioral therapy (CBT) for premenstrual syndrome and PMDD [11] reported that Acceptance and Commitment Therapy (ACT) [12] may be well-suited to treat the characteristics of PMS/ PMDD.

Recently, ACT, which aims to enhance psychological flexibility, has demonstrated effectiveness in improving emotion regulation and fostering positive parent-child relationships. While ACT is part of traditional CBT, it specifically focuses on the functions and contexts of psychological phenomena rather than the content of thoughts, feelings, and sensations. The ACT, established by Hayes et al., comprises six core therapeutic processes: “contacting the present moment,” “defusion,” “acceptance,” “self-as-context,” “values,” and “committed action”. ^12^ A systematic review of ACT’s effectiveness in supporting various groups of parents, including those raising children with chronic pain, revealed improvements in parent-reported symptoms of their children’s physical or psychological functioning, as well as reductions in parent-reported measures of stress, depression, and anxiety [12–20].

Research indicates that parents with low psychological flexibility are more likely to employ ineffective parenting practices, such as harsh discipline and inconsistent rules [21, 22]. Brassell et al. [23] examined the relationships between parental psychological flexibility, adaptive parenting practices, and children’s internalizing and externalizing problems at three developmental stages (toddler age; 3–7 years, childhood age; 8–12 years, adolescence age; 13–17 years). The results showed that higher parent-specific psychological flexibility was indirectly associated with lower levels of children’s internalizing and externalizing problems through adaptive parenting practices across children’s age groups. In the toddler model of that study, parent role-related psychological flexibility strongly influenced adaptive parenting practices, which were constructed with positive and negative parenting, and harsh and lax discipline practice. Mayor et al. [24] conducted 60-minute ACT sessions four times a week and found that the sessions improved parents’ positive parenting behaviors and maintained them after six weeks. Fonsenca et al. [25] revealed that parenting stress directly and indirectly affected parenting style through psychological flexibility within parenting and that low psychological flexibility in parenting was associated with low use of democratic parenting styles and high use of authoritarian parenting styles. These studies suggest that parental psychological flexibility is strongly associated with positive parenting and negative parenting and that psychological flexibility may function as a buffering factor.

**Fig. 1:**
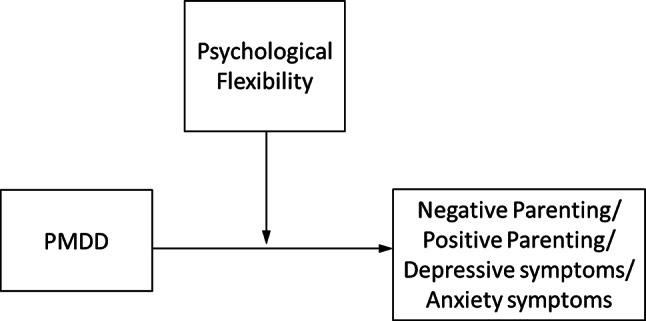
Adjusted hypothesis model of the relationship between PMDD and Negative Parenting (Depressive or Anxiety Symptoms) by Psychological Flexibility

Based on these findings, PMDD is believed to have an impact on symptoms of depression, anxiety, and parenting behaviors. Although the relationship between PMDD and psychological flexibility remains unclear, we hypothesize that psychological flexibility may moderate the effects of PMDD on depression, anxiety, and parenting behaviors (see Fig. 1). If psychological flexibility is shown to alleviate PMS/PMDD symptoms and reduce negative parenting behaviors, along with depressive and anxiety symptoms, it would highlight the utility of ACT in supporting parents and would clarify how to expand and adapt these interventions.

## Method

### Participants

Data for this secondary analysis were collected as part of research on the standardization of the Japanese version of the Parental Acceptance and Action Questionnaire (PAAQ-J) in December 2020 [26]. The participants were recruited by Rakuten Research, Inc., an online marketing research company with access to approximately 2.3 million Japanese survey respondents. An e-mail containing a link to an online questionnaire was sent to randomly selected individuals stratified by sex and age across Japan.

The sample comprised 2,000 mothers raising children aged 0 to 3 years (mean age of children: 1.57 ± 0.74 years; mean age of mothers: 33.58 ± 4.7 years), with 500 children in each age category (0, 1, 2, and 3 years). The inclusion criterion required participants to be mothers of children aged between 0 and 3 years, with no exclusion criteria applied. If a mother had more than one child within the specified age range, she was asked to select one child for the survey.

### Measures

#### Demographic information

The mothers were asked to provide demographic information, including their age, current medical history (mental disorders, physical diseases, PMS, and PMDD), ongoing medications, menstruation details (including premenstrual, menstrual, and postmenstrual phases), menstrual cycle characteristics, menstrual stability, employment status, and number of births. Additionally, information about the children was collected, including the number of children, eligible children’s ages, and the presence or absence of disabilities.

#### Japanese version of the parent acceptance and action questionnaire (PAAQ-J )

Originally developed by Cheron et al. [27] with the Japanese version developed by Okajima and Okajima [26], the Parental Acceptance and Action Questionnaire (PAAQ-J) is a scale to assess caregivers’ psychological flexibility regarding experiential avoidance. It is a scale comprising 12 items across three factors: inaction behavior, inaction perception, and unwillingness (Cronbach’s α = .80 for the total scale; α = .84, .72, and .68 for each factor, respectively). The items were responded to using a self-administered 7-point (1 = Never True to 7 = Always True) Likert scale. Test-retest reliability showed an intraclass correlation coefficient of .49 (95% CI: .44–.54). Higher scores on the PAAQ-J indicate greater psychological inflexibility [28].

#### Hospital anxiety and depression scale (HADS)

The HADS is a 14-item scale assessing anxiety and depressive symptoms, comprising seven items each for anxiety (HADS-A) and for depression (HADS-D). Participants respond to each symptom on a 4-point scale (1–4), with higher scores indicating more severe symptoms. The Japanese version of the HADS was developed by Hatta et al. [29], with a Cronbach’s α of .80 for HADS-A, and between .59 and .61 for HADS-D. Higher HADS scores reflect greater levels of depression and anxiety.

#### Premenstrual dysphoric disorder scale (PMDDS)

The PMDDS, a 17-item scale measuring symptoms of PMDD, includes a four-item self-report questionnaire (1 = Not at all to 4 = Very much), with higher scores indicating more severe PMDD symptoms. The scale encompasses six items related to fatigue and/or physical symptoms, seven for depressive moods, and four for dysfunctional relationships and/or anger. An individual is suspected of PMDD when they endorse at least one item with a score of 4 (Very much) and at least four items with a score of 3 (Quite a lot) or 4 (Very much). Cronbach’s alpha coefficients were α = .85 for fatigue and/or physical symptoms, α = .84 for depressive moods, α = .84 for dysfunctional relationships and/or anger, and α = .77 for the total PMDDS.

#### Questionnaire on dealing with children (QDC)

The QDC is an eight-item scale that assesses children’s coping behavior and comprises two factors: five items related to negative parenting and three related to positive parenting. The responses were rated a four-point scale (1 = Not at all to 4 = Very much). Cronbach’s α coefficients were .79 for negative parenting, .71 for positive parenting, and .81 for the total QDC.

### Procedures

This study was approved by the Research Ethics Committees of the University of Tokyo Health Sciences (17–27 H) and Rikkyo University (21–31).

### Data analysis

First, correlation analysis was conducted to examine the relationships among the scales. Correlation coefficients were interpreted based on Cohen’s criteria [30], with *r* =.1 indicating a small effect, *r* =.3 a moderate effect; and *r* =.5 a large effect. Second, a hierarchical multiple regression analysis was performed to assess the moderating effect of the PAAQ on the relationships between PMDDS and HADS-A, HADS-D, positive parenting, and negative parenting. In this analysis, PMDD served as the independent variable, while HADS-A, HADS-D, positive parenting, and negative parenting were the dependent variables. The PAAQ was included as a moderating variable, and demographic data were controlled. Interaction terms were created using centered variables and were subsequently added to the model. If the interaction effect was significant, a simple slope test was conducted to elucidate the nature of the moderation effect. Statistical analyses were conducted using SPSS Statistics version 27 (IBM Corp., Armonk, NY, USA) and R statistical software version 4.4.0 (R Project for Statistical Computing, Vienna, Austria).

## Results

### Demographic data

The analysis included 1,538 menstruating mothers (227 with 0-year-old children, 428 with one-year-olds, 409 with two-year-olds, and 424 with three-year-olds) out of an initial sample of 2,000. Parent and child demographic data, including the number of children, employment status, birth order, and child age, are summarized in Table 1.

**Table 1:**
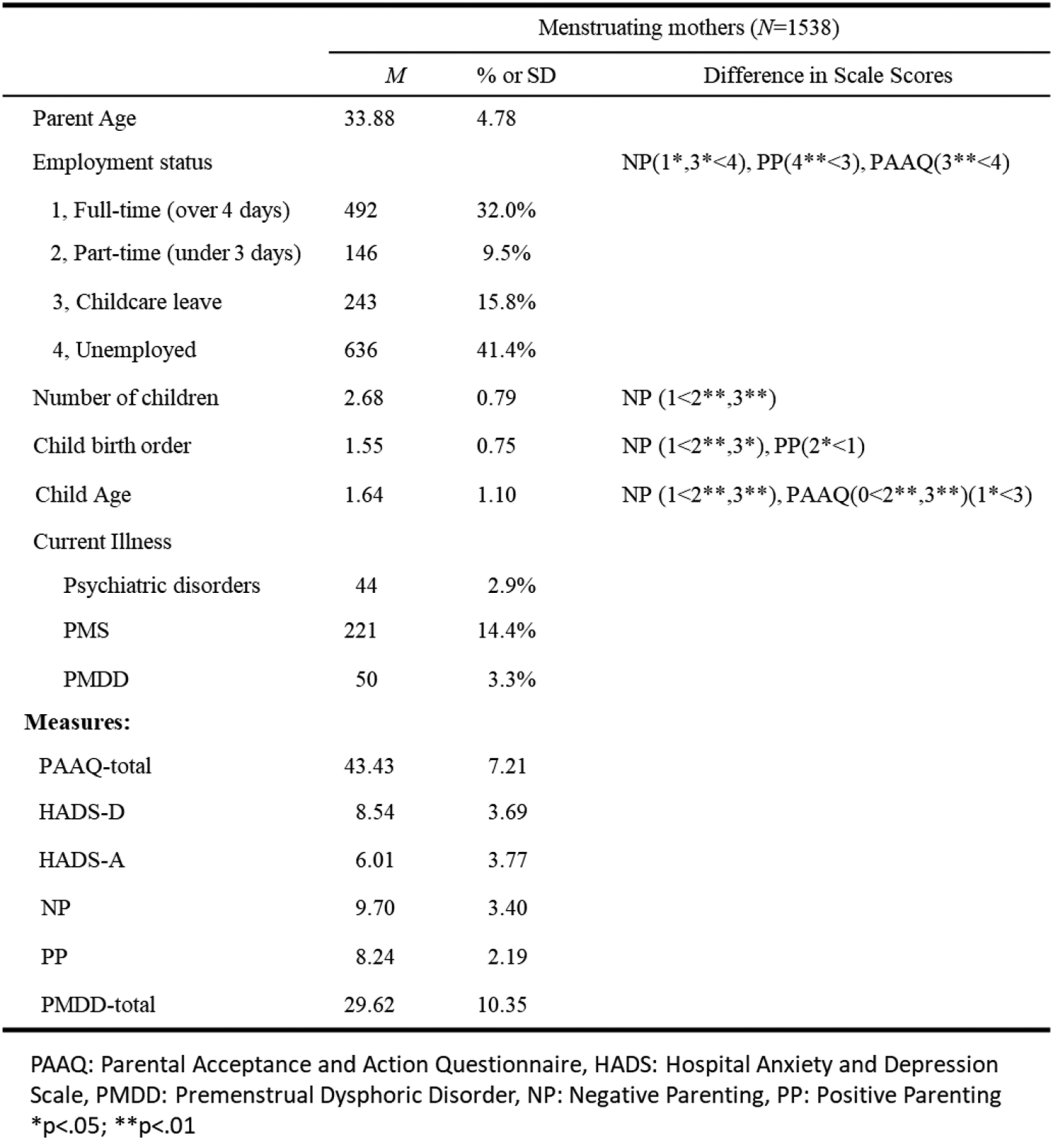
Sample demographic characteristics and mean of each variable (standard deviations are in parentheses) and difference in Scale Scores

One-way ANOVA was conducted to examine differences in scale scores (PAAQ, HADS-D, HADS-A, negative parenting, and positive parenting) based on parent and child attributes (number of children, employment status, birth order, and child age). Scale scores were subjected to multiple comparisons using Bonferroni correction wherever significant differences were identified. For negative and positive parenting, we focused on parents with children aged 1–3 years, as the questionnaire items did not apply to children aged 0. The results indicated no significant differences in HADS-D, HADS-A, or PMDD scores based on parent or child attributes. Differences in PAAQ, negative parenting, and positive parenting scores related to these attributes are presented in Table 1.

### Correlation coefficients among each scale

Correlation analysis revealed significant correlations among all scales (Table 2). The PMDD was positively and moderately correlated with PAAQ (*r* =.31), HADS-D (*r* =.30), HADS-A (*r* =.54), and negative parenting (*r* =.34). Additionally, PAAQ scores exhibited a positive and moderate correlation with negative parenting (*r* =.44).

**Table 2:**
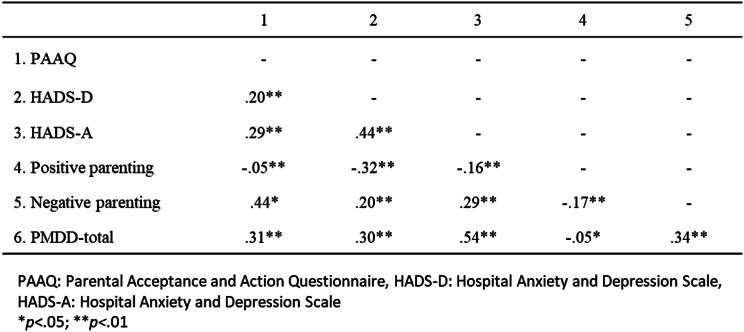
Pearson’s correlation coefficient between each scale in this study at *N*=1538

### Moderation analysis

Hierarchical multiple regression analysis was conducted to investigate the moderating effect of PAAQ on the relationship between PMDD and HADS-A, HADS-D, negative parenting, and positive parenting. In the analysis, the PMDD served as the independent variable, while HADS-A, HADS-D, negative parenting, and positive parenting were treated as dependent variables. The PAAQ acted as the moderating variable, with demographic data (number of children, employment status, birth order, and child age) controlled for in the analysis. Interaction terms were created using centrally processed variables and subsequently included in the model.

The results from the hierarchical multiple regression analysis (Tables 3 and 4) indicated that for HADS-D, the *R*^2^ at step 3 was significant (*R*^2^ = .102, *F* = 34.838, *p* < .001); however, the change in *R*^2^ (*ΔR*^2^) was not significant, suggesting the interaction term did not contribute additional explanatory power. For the HADS-A, *R*^2^ at step 3 was significant (*R*^2^ = .311, *F* = 138.476, *p* <.001) and the *ΔR*^2^ was also significant (*ΔR*^2^ = .004, *p* <.01). In the case of positive parenting, the *R*^2^ at step 3 was significant (*R*^2^ = .023, *F* = 6.011, *p* <.001) and the *ΔR*^2^ was also significant (*ΔR*^2^ = .008, *p* <.001). The interaction term between PMDD and PAAQ revealed a significant standardized partial regression coefficient (*β* = .498, *p* <.001). Conversely, for negative parenting, the *R*^2^ at Step 3 was significant (*F* = 78.593, *p* <.001), but the *ΔR*^2^ was not.

**Table 3:**
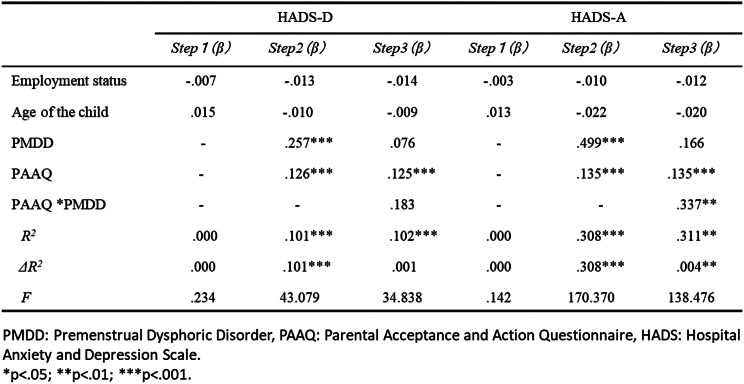
Hierarchical multiple regression analysis with depression, anxiety as objective variables (*N*=1538)

**Table 4:**
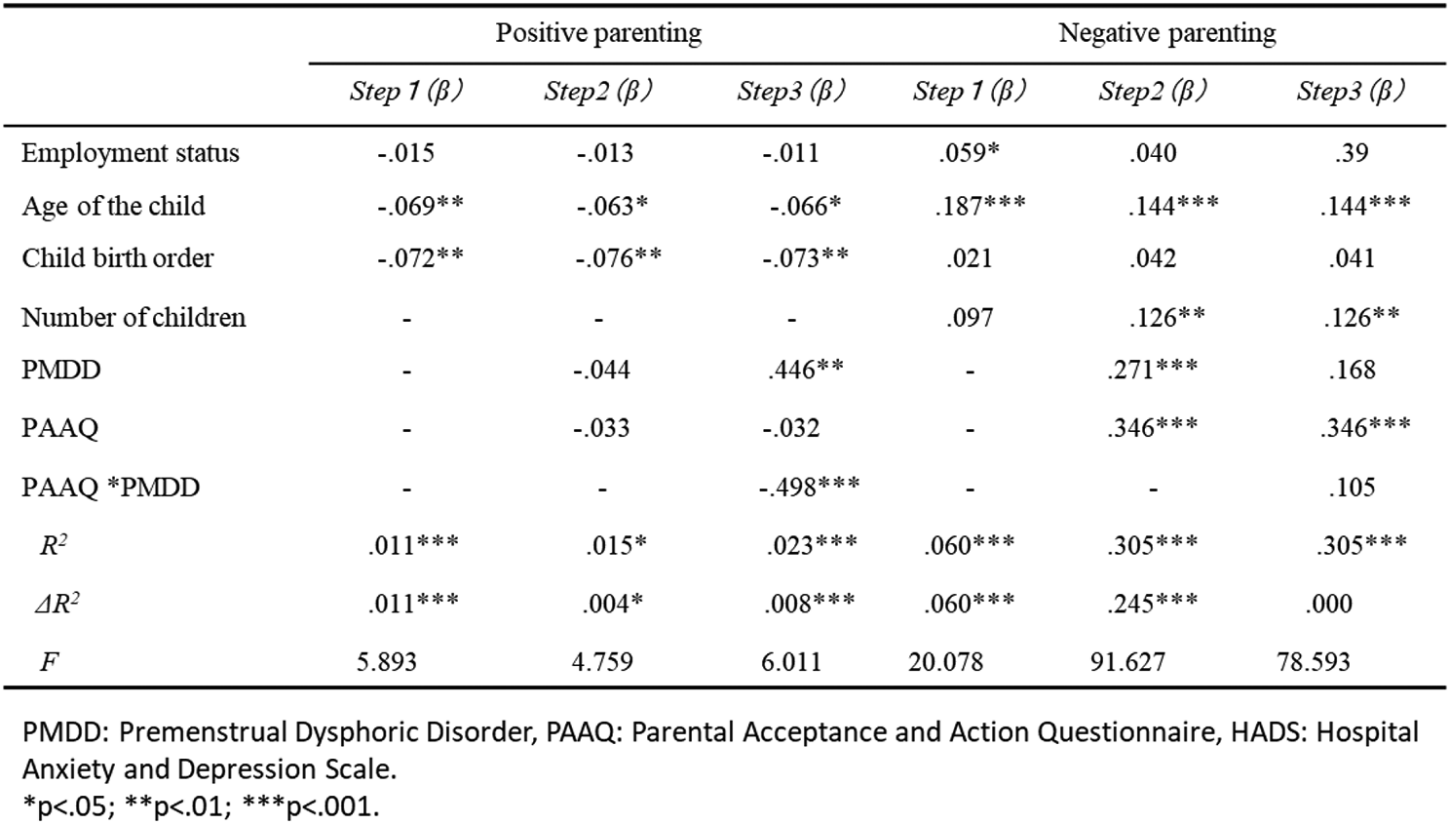
Hierarchical multiple regression analysis with Positive parenting, Negative parenting as objective variables (*N*=1261)

**Fig. 2:**
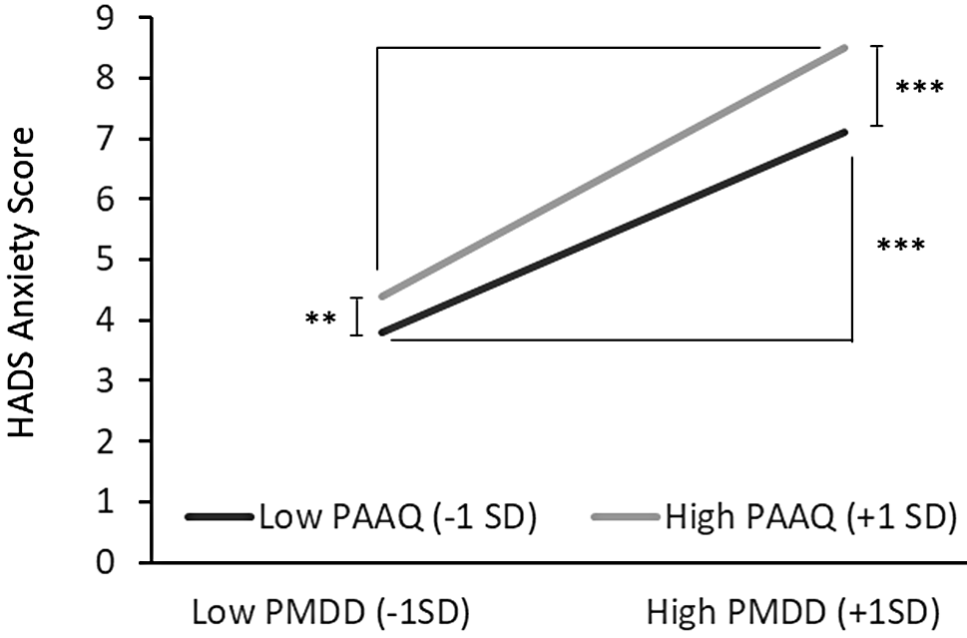
Simple regression line (PMDD) by PAAQ group in Anxiety

Given the significant standardized partial regression coefficients for the interaction between PMDD and PAAQ in relation to HADS-A, a simple slope analysis was performed (Fig. 2). This analysis illustrated the regression of PMDD on HADS-A at each respective value of PAAQ, utilizing + 1*SD* and − 1*SD* as reference points. The findings indicated that lower PMDD scores were associated with lower HADS-A, while higher PMDD correlated with higher HADS-A for both good PAAQ (-1*SD*) and poor PAAQ (+ 1*SD*) (simple slope = .16, *p* <.001; simple slope = .20, *p* <.001). Furthermore, lower PMDD was linked to lower HADS-A scores when PAAQ was better; similarly, even in cases of high PMDD, HADS-A scores were lower with better PAAQ (*simple slope* = .04, *p* <.01; *simple slope* = .10, *p* < 001).

**Fig. 3:**
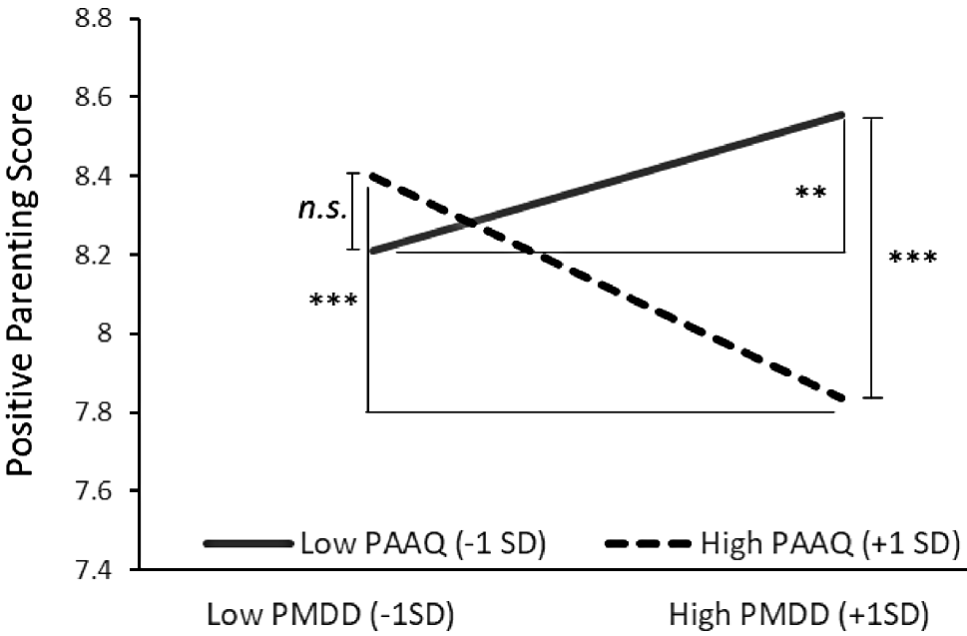
Simple regression line (PMDD) by PAAQ group in Positive parenting

Since positive parenting also demonstrated significant standardized partial regression coefficients for the interaction, a simple slope analysis was conducted (Fig. 3). This analysis illustrated the regression of PMDD on positive parenting at each respective value of PAAQ, again using + 1 *SD* and − 1 *SD* as the standard. Further, lower PMDD was associated with higher positive parenting when PAAQ was good (-1 *SD*) (*simple slope* = .02, *p* < 05). Conversely, for poorer PAAQ (+ 1*SD*), lower PMDD was related to higher positive parenting, while higher PMDD was also linked to higher positive parenting (*simple slope* = -.03, *p* < 001). For low PMDD, there was no significant difference in positive parenting between good and poor PAAQ; however, for high PMDD, good PAAQ was associated with high positive parenting while poor PAAQ correlated with low positive parenting (*simple slope* = .01, *n.s.*; *simple slope* = -.05, *p* < 001).

## Discussion

This study aimed to determine whether psychological flexibility acts as a moderating factor in the effects of PMDD symptoms on negative parenting, positive parenting, depressive symptoms, and anxiety symptoms in mothers of children aged 0–3 years. The results showed that psychological flexibility moderates the effects of PMDD symptoms on anxiety and positive parenting behaviors and that psychological flexibility has a direct effect on depression and negative parenting behaviors, rather than a moderating effect.

From the initial sample of 2,000 mothers raising children between the ages of 0 and 3 years, the analysis focused on 1,538 menstruating mothers. For the analysis of negative and positive parenting behaviors, we further narrowed the sample to 1,261 parents of children aged 1–3 years, as the item content related to parenting did not apply to 0-year-old children. We examined the differences in scale scores (PAAQ, HADS-D, HADS-A, PMDD, negative parenting, and positive parenting) across various parent-child attributes, including the number of children, employment status, birth order, and child age.

The results exhibited no significant differences in depression, anxiety, or PMDD symptoms based on these attributes. However, differences were found in negative parenting, positive parenting, and psychological flexibility. Specifically, psychological flexibility was lower among unemployed parents compared to those on parental leave, and it was also lower among parents raising two- and three-year-olds compared to those with younger children. Additionally, parents on parental leave or in full-time employment, those raising one child, and those raising younger children were less likely to engage in negative parenting behaviors. Parents on parental leave were more likely to engage in positive parenting than unemployed parents, and those raising their second child were more likely to engage in positive parenting than those raising their first child.

In Japan, several studies have highlighted the parenting stress experienced by mothers raising infants and toddlers [31]. Research has shown that unemployed mothers often feel more isolated and are more prone to losing their sense of identity [32]. Additionally, mothers raising two- and three-year-olds report greater stress related to managing anger and aggression toward their children compared to mothers of one-year-olds [33]. This heightened stress may stem from children entering the “terrible twos” or primary rebellious period, during which their behavior becomes more defiant and argumentative [34]. Consequently, mothers’ psychological flexibility may decrease, making them more prone to engaging in negative parenting behaviors.

Based on these findings, mothers in Japan are more likely to increase their psychological flexibility, engage in less negative child-rearing behaviors, and in more positive child-rearing behaviors when they have social ties, such as child-rearing leave, compared to when they are unemployed. The number and age of children are also very important in this matter, and mothers raising two or three children or raising two- or three-year-olds are more likely to engage in negative parenting behaviors and need more support.

Pearson’s correlation coefficients were calculated for each scale score: the HADS-D, HADS-A, PAAQ, negative parenting, positive parenting, and PMDD-total. 25), negative parenting and the PAAQ (*r* =.44), and HADS-A and the PAAQ (*r* =.54) showed moderate positive correlations. Miyaoka et al. [35] surveyed menstruating 20- to 45-year-olds (of whom 37% were women with children) and found a moderate positive correlation (*r* =.54) between PMDD symptoms and depression, which is consistent with the results of this study. Brassell et al. [23] in a study of 210 subjects raising 3-5-year-olds found moderate (*r* =.40) and weak (*r* =.25) correlations between the AAQ-II, which measures psychological flexibility in general, not just parenting, negative parenting, and positive parenting. Fonseca et al. [25] studied 250 mothers with children aged 2–12 years and found moderate correlations between the AAQ-II and anxiety and depression (*r* =.43, *r* =.47) and moderate correlations with a parenting style that uses corporal punishment (authoritative) (*r*=-.46). For the PAAQ, which measures psychological flexibility related to parenting used in this study, the moderate correlation with negative parenting is consistent with previous studies. In contrast, positive parenting was not correlated with the PAAQ in the present study, whereas in a previous study [23], permissive parenting style and AAQ-II were weakly correlated. This may be because in positive parenting, given that PAAQ functioned as a regulating variable, there were many surplus variables, although related, that did not show up in the correlation.

In this study, we examined whether the PAAQ functions as a moderating variable for PMDD and depression, anxiety, and positive and negative parenting and found that it is a moderating variable for anxiety and positive parenting. When PMDD symptoms were high, psychological inflexibility resulted in higher anxiety and less positive parenting. When PMDD symptoms were low and psychological inflexibility was present, anxiety was low, and Positive Parenting was increased. Additionally, for those with low PMDD symptoms, positive parenting was not influenced by psychological flexibility, whereas for those with high PMDD symptoms, it was highly influenced by psychological flexibility; if inflexible, positive parenting was significantly low. This indicates that even mothers with PMDD symptoms can be positively involved with their children if they have high psychological flexibility. The finding highlights the effect of ACT for parents with PMDD. The commitment component of ACT involves teaching patients how to recognize and live according to their values or personal commitments [12]. Lustyk et al. [11] reported that the natural, biological process of the menstrual cycle in healthy women is recurrent, predictable, and will have symptoms associated with the ebb and flow of the hormones which drive it; therefore, learning acceptance of this process and ways of coping amidst these changes would be more beneficial than focusing on assessment and modification of irrational thinking. Thus, parents with greater psychological flexibility might be able to engage positively in parenting without being influenced by PMDD symptoms.

Unlike anxiety and positive parenting, however, no moderating effect of psychological flexibility was found for depression and negative parenting, although direct effects were confirmed. It is unclear why psychological flexibility does not have a buffering effect on depression and negative parenting from the results of this study. In the future, it will be necessary to examine other factors that may alleviate these symptoms and behaviors in mothers with PMDD.

The findings of this study further suggest that greater psychological flexibility may help reduce anxiety and promote positive parenting, even among mothers with high PMDD symptoms. Given that approximately 18% of the mothers in this study were diagnosed with PMDD or PMS, providing psychological support to enhance psychological flexibility is crucial, as one in five mothers is affected by hormonal fluctuations.

In Japan today, possible facilities for working with mothers raising children aged 0–3 include day-care centers, local childcare centers, and gynecology departments for the treatment of PMDD. Among them, obstetrics and gynecology departments that provide one-month checkups, pediatrics departments that provide checkups for 0-year-olds, and health centers that provide checkups for children1-6 months and three-year-olds may be more likely to offer programs related to mothers’ mental health. In the future, early intervention and prevention programs using ACT programs should be based on empirical studies to increase psychological flexibility and prevent negative parenting.

### Limitations

The first limitation of this study is its cross-sectional design, which limits the ability to infer causal relationships. While the direction of associations explored in the adjustment model aligns with prior literature, the influence of psychological flexibility and its moderating effects were only examined within this framework. Without testing these relationships in a longitudinal design, definitive conclusions about causality cannot be drawn. Future longitudinal studies on PMDD symptoms, psychological flexibility, nurturing behaviors, and emotional problems are essential for a clearer understanding of these dynamics.

A second limitation is the exclusive focus on mothers. The emotional problems of mothers have been linked to their relationships with their partners, their support systems, and their children’s developmental characteristics. Future studies should investigate whether mothers of children with developmental disorders, such as autism, fit within the model used in this study. This would require controlling for factors such as marital relationships, support networks, and other contextual influences.

A third limitation is the low Cronbach’s α coefficient for the depression subscale of the HADS (*α* = 0.59-0.61). While some researchers in the social sciences consider a Cronbach’s *α* above 0.60 acceptable [36], the recommended threshold is generally 0.70 or higher. Therefore, caution is warranted when interpreting these results. The HADS was used in this study because it measures both anxiety and depression, but future research should consider using scales with higher reliability and validity when assessing depressive symptoms.

## Conclusion

The results of this study suggest that increasing the psychological flexibility of mothers with PMDD symptoms who are raising infants and toddlers would have a positive effect in terms of decreasing anxiety and increasing positive parenting behavior. Furthermore, a strong, direct effect of psychological flexibility was shown for depression and negative parenting for parents with/without PMDD symptoms. Future studies will be necessary to further clarify the moderating effect of psychological flexibility. Moreover, to gain a deeper understanding of the potential benefits of psychological flexibility, it will be necessary to conduct intervention studies based on ACT for mothers with PMDD who are raising children to verify its effectiveness and investigate influencing factors.

## Data Availability

Data utilized in this study is available upon request from the corresponding author.

## Abbreviations

PMDD: Premenstrual Dysphoric Disorder
PMS: Premenstrual Syndrome
HADS: Hospital Anxiety and Depression Scale
HADS: D–Hospital Anxiety and Depression Scale (Depression subscale)
HADS: A–Hospital Anxiety and Depression Scale (Anxiety subscale)
PAAQ: Parental Acceptance and Action Questionnaire
ACT: Acceptance and Commitment Therapy
AAQ: II–Acceptance and Action Questionnaire–II
